# Validation of Two Screening Tools for Anxiety in Hemodialysis Patients

**DOI:** 10.3390/jpm12071077

**Published:** 2022-06-30

**Authors:** Els Nadort, Noëlle J. K. van Geenen, Robbert W. Schouten, Rosa E. Boeschoten, Prataap Chandie Shaw, Louis Jean Vleming, Marcel Schouten, Karima Farhat, Friedo W. Dekker, Patricia van Oppen, Carl E. H. Siegert, Birit F. P. Broekman

**Affiliations:** 1 Department of Psychiatry, OLVG Hospital, Jan Tooropstraat 164, 1061 AE Amsterdam, The Netherlands; n.j.k.vangeenen@olvg.nl (N.J.K.v.G.); b.f.p.broekman@olvg.nl (B.F.P.B.); 2 Department of Psychiatry, Amsterdam University Medical Centre and GGZ inGeest, Oldenaller 1, 1081 HJ Amsterdam, The Netherlands; r.boeschoten@ggzingeest.nl (R.E.B.); p.vanoppen@ggzingeest.nl (P.v.O.); 3 Department of Nephrology, OLVG Hospital, Jan Tooropstraat 164, 1061 AE Amsterdam, The Netherlands; r.schouten@olvg.nl (R.W.S.); c.siegert@olvg.nl (C.E.H.S.); 4 Department of Nephrology, Haaglanden Medisch Centrum, Lijnbaan 32, 2512 VA The Hague, The Netherlands; p.chandieshaw@haaglandenmc.nl; 5 Department of Nephrology, HagaZiekenhuis, Els Borst-Eilersplein 275, 2545 AA The Hague, The Netherlands; l.vleming@hagaziekenhuis.nl; 6 Department of Nephrology, Tergooi Ziekenhuis, Van Riebeeckweg 212, 1213 XZ Hilversum, The Netherlands; maschouten@tergooi.nl; 7 Department of Nephrology, Spaarne Gasthuis, Boerhaavelaan 22, 2035 RC Haarlem, The Netherlands; kfarhat@spaarnegasthuis.nl; 8 Department of Clinical Epidemiology, Leiden University Medical Centre, Albinusdreef 2, 2333 ZA Leiden, The Netherlands; f.w.dekker@lumc.nl

**Keywords:** anxiety disorders, mass screening, renal dialysis

## Abstract

Background: Symptoms of anxiety are often unrecognized and untreated in dialysis patients. We investigated the diagnostic accuracy of two widely used screening tools for anxiety in hemodialysis patients. Methods: For this cross-sectional validation study, chronic hemodialysis patients from eight dialysis centers in the Netherlands were included. The Beck Anxiety Inventory (BAI) and Hospital Anxiety and Depression Scale—Anxiety subscale (HADS-A) were validated by the Mini International Neuropsychiatric Inventory (MINI) diagnostic interview. Receiver operating characteristic curves were used to determine the optimal cut-off values. Results: Of 65 participants, 13 (20%) were diagnosed with one or more anxiety disorders on the MINI, of which 5 were included in the analysis. ROC curves showed a good diagnostic accuracy of the BAI and HADS-A. The optimal cut-off value for the BAI was ≥13 (sensitivity 100%, specificity 85%) and for the HADS-A was ≥10 (sensitivity 80%, specificity 100%). Conclusions: Based on our limited data, both the BAI and the HADS-A seem to be valid screening instruments for anxiety in hemodialysis patients that can be used in routine dialysis care. The HADS-A consists of fewer items and showed fewer false-positive results than the BAI, which might make it more useful in clinical practice.

## 1. Introduction

Anxiety is characterized by excessive fear that can cause clinically significant distress or impairment of functioning. Excessive anxiety can begin without a clear reason (panic disorder), can be triggered by a traumatic event or situation (post-traumatic stress disorder (PTSD)), can be due to a fear of social or performance situations (social anxiety disorder), can be triggered by the presence or anticipation of a specific object or situation (specific phobia), or can be due to a number of events or activities (general anxiety disorder (GAD)) [1].

Recently, the nephrology field has become aware that elevated anxiety symptoms are a common problem in dialysis patients, with a prevalence of 19–43% and with a large impact on quality of life and adverse clinical outcomes such as impaired treatment adherence, hospitalization, and mortality [2,3,4,5,6]. Due to the overlap of symptoms of anxiety with symptoms of other medical conditions, such as depression and uremia, symptoms of anxiety are often unrecognized and untreated in dialysis patients [2,6]. Furthermore, international nephrology guidelines inadequately address screening for anxiety, and no recommendations on the frequency and preferred screening tools have been proposed [7]. Studies of chronic kidney disease and cardiovascular disease patients investigating treatments for anxiety have demonstrated that the results of psychotherapeutic interventions are promising in both lowering symptoms of anxiety as well as reducing clinical outcomes such as mortality [8,9,10]. To identify dialysis patients who might be in need of treatment for anxiety, validated anxiety screening instruments that can easily be applied in routine dialysis care are needed [2].

Although there are various screening tools for anxiety available, only a few of those have been validated in hemodialysis patients [11,12]. A well-established screening instrument for anxiety is the Beck Anxiety Inventory (BAI) [13,14]. The BAI was developed to assess the severity of anxiety while minimizing the overlap with depression [13]. The BAI has been used extensively, and has been validated in medical settings as well as in older adults [15,16,17,18,19,20]. To our knowledge, the BAI has not yet been validated in dialysis patients. The Hospital Anxiety and Depression Scale (HADS) is shorter than the BAI, and was developed as a self-assessment screening tool for the detection of the presence of anxiety and depressive disorders specifically for adults attending medical outpatient clinics [21]. The HADS excludes somatic symptoms of anxiety and depression that are common in medical patients related to physical illness. The HADS has been used extensively and was found to perform well in other somatic patients, although evidence in dialysis patients has been inconclusive [11,12,17,22,23]. Two studies found acceptable performance and recommended the use of the HADS—Anxiety subscale (HADS-A) in dialysis patients; however, another study found poor predictive power of the HADS-A [11,12,23]. As diagnostic accuracy of screening tools varies between settings and patient groups, further validation is needed in hemodialysis patients.

This study aimed to investigate the diagnostic accuracy of two widely used screening tools for anxiety, the BAI and HADS-A, and validate these screening tools against a structured psychiatric diagnostic interview for detecting clinically relevant symptoms of anxiety in hemodialysis patients.

## 2. Materials and Methods

### 2.1. Study Design and Population

To validate the BAI and HADS-A in dialysis patients, baseline data were used from the ongoing multicenter Depression Related Factors and Outcomes in Dialysis Patients With Various Ethnicities and Races Study—Internet Intervention (DIVERS-II), which consists of a randomized controlled trial (RCT) and a parallel observational cohort. The extensive study protocol has been published previously [24]. In short, the RCT of DIVERS-II is investigating the effectiveness of online self-help intervention for depressive symptoms in hemodialysis patients. Patients who could not be randomized due to low depression scores were offered to participate in the parallel observational cohort study. Consecutive patients who gave informed consent for participation in both the RCT and observational cohort of the DIVERS-II study between 2 October 2020 and 10 February 2021 were asked to participate in this validation study. Adult patients from 8 dialysis centers affiliated with 5 hospitals in the Netherlands receiving maintenance hemodialysis (>90 days) without severe psychiatric pathology, who were able to read or understand Dutch, and were willing to undergo a psychiatric diagnostic interview were included in this validation study. The study protocol, information brochure, and informed consent were approved by the Medical Ethics Committee of MEC-U, Nieuwegein, the Netherlands (registration number: NL58520.100.17). This study was approved by the medical ethics committees of all participating hospitals (Supplementary Table S1). The study was carried out in accordance with the Declaration of Helsinki, and written informed consent was obtained from all participants before participation. The study was prospectively registered in the Dutch Trial Register (Trial NL6648). This study was carried out in accordance with the STARD 2015 reporting guideline for diagnostic accuracy studies [25].

### 2.2. Anxiety Screening Tools

Symptoms of anxiety were measured with the BAI and the HADS-A, the most frequently used screening tools for assessing anxiety symptoms in chronic kidney disease patients [5]. The BAI consists of 21 items related to common somatic and cognitive symptoms of anxiety in which respondents are asked how much these symptoms have bothered them in the past week, on a scale ranging from 0 (not at all) to 3 (severely). The total score is between 0 and 63, where higher scores indicate more severe anxiety [14]. The HADS-A is a subscale of the HADS and consists of 7 items on anxiety, on which patients are asked about the frequency or severity of this item in the past week on a scale ranging from 0 (never) to 3 (almost always). The HADS-A total score ranges between 0 and 21, with higher scores indicating more severe anxiety [21]. The BAI takes approximately 5 min to complete and the HADS-A approximately 2 min.

### 2.3. Reference Standard

The scores of the BAI and the HADS-A were compared to a *Diagnostic and Statistical Manual of Mental Disorders,* Fourth Edition (DSM-IV) diagnosis of an anxiety disorder, determined by using the latest version of the Mini International Neuropsychiatric Inventory (MINI; 5.0.0 Dutch version) [1,26]. The MINI is a widely used structured psychiatric diagnostic interview instrument and is considered a reference standard diagnostic tool. We used sections for anxiety disorders (panic disorder, agoraphobia, social phobia, specific phobia, PTSD, and GAD) and sections for mood disorders (depressive episodes and dysthymia). The sections on depression were used to aid in the diagnosis of GAD, as this can only be diagnosed if depression is ruled out. If patients with a specific phobia did not have an encounter with the object or situation of their phobia in the past two weeks, they were excluded from the analysis, as these patients were unlikely to have experienced anxiety that could be measured by the BAI or the HADS-A, which measure symptoms experienced in the past week.

The MINI interviews were administered by a medical resident with clinical experience in psychiatric care within one week after the self-reported scales were filled out, during a dialysis session (62 interviews) or over the telephone due to COVID-19 (3 interviews). The administration time of the MINI was 15 to 45 min. The medical resident was trained by a supervising psychiatrist with extensive experience with MINI interviews. All MINI interviews were reviewed by the supervising psychiatrist, and 10 random MINI interviews were performed by both the medical resident and the psychiatrist to assess interrater reliability. To minimize rating bias by knowledge of the self-reported scales, the interviewer was blinded to the scores of the self-reported scales.

### 2.4. Data Collection

At baseline, sociodemographic and clinical data were collected through self-reported questionnaires and electronic patient files. The primary cause of kidney disease was classified according to the European Renal Association–European Dialysis and Transplant Association (ERA-EDTA) coding system and divided into four groups (renal vascular disease, diabetic nephropathy, glomerulonephritis, and other) [27]. The Davies comorbidity index was used to define the level of comorbidity [28].

### 2.5. Power Calculation

A total sample size of 60 participants was required when selecting a sensitivity of 98% and specificity of 85%, with a clinically acceptable width of no larger than 10% for sensitivity and specificity of the 95% confidence level when accounting for the estimated dropout rate of 5% and estimated prevalence of 22% in this cohort [29].

### 2.6. Statistical Analysis

Standard descriptive statistics were used to present the baseline characteristics of the study population, depending on the variable and underlying distribution. Inter-rater reliability was calculated with the kappa statistic and interpreted using the guidelines for the strength of agreement from Landis and Koch [30].

The unidimensionality of the BAI and HADS-A was analyzed in a 1-factor model using confirmatory factor analysis (CFA) with robust full-information maximum likelihood estimation. Model fit was interpreted by inspecting the comparative fit index (CFI) with an acceptable fit if greater than 0.900 and the root-mean-squared error of approximation (RMSEA) with a good fit if less than 0.060. CFA was performed in R (R Core Team) using the package lavaan [30,31]. Chronbach’s alpha was calculated to provide a measure of internal consistency. 

To determine the diagnostic accuracy of the BAI and HADS-A, receiver operating characteristic (ROC) curves were plotted and the area under the curve (AUC) was determined. The optimal cut-off score was assessed using the highest Youden Index [32]. In addition, sensitivity, specificity, positive predictive value (PPV), negative predictive value (NPV), positive likelihood ratio (LR+), and negative likelihood ratio (LR-) were calculated for the optimal cut-off scores. Statistical analyses were performed using SPSS for Windows, version 21 (IBM Corp).

## 3. Results

### 3.1. Participants and Baseline Characteristics

**Figure 1:**
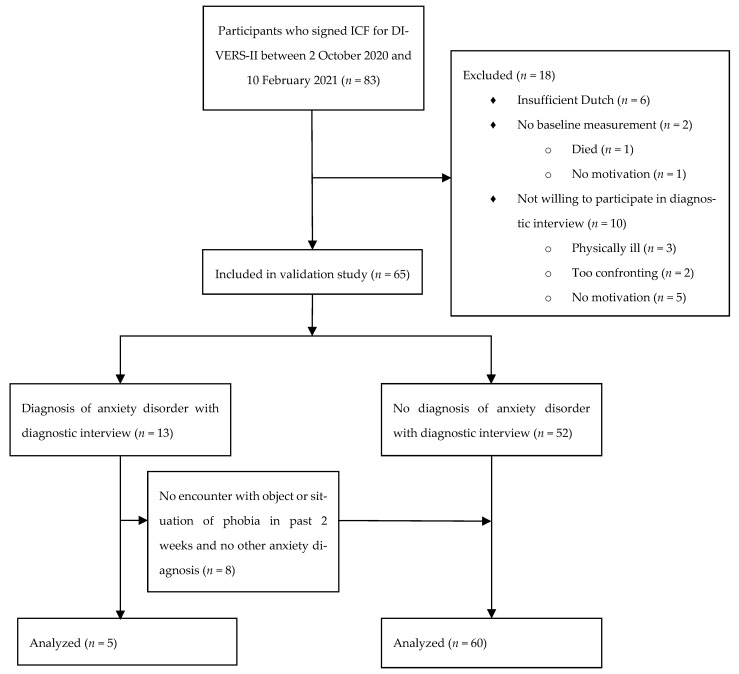
Patient flow diagram. Abbreviations: ICF, informed consent form; DIVERS-II, Depression Related Factors and Outcomes in Dialysis Patients With Various Ethnicities and Races Study—Internet Intervention.

Participant flow is shown in Figure 1. Baseline characteristics are presented in Table 1. We included a total of 65 patients, of which 69% were male with a mean age of 66 (standard deviation (SD) 13) years. The mean dialysis vintage was 23 months (interquartile range (IQR) 8–38). The majority of patients (62%) had a moderate Davies comorbidity score, and almost half of the patients (45%) had diabetes mellitus as a comorbid condition.

In the medical history, 6% had a diagnosis of major depressive disorder, and none of the patients had an anxiety disorder. At baseline, one patient was currently treated with psychotherapy, and 10 patients (16%) were using antidepressants. The mean baseline BAI score was 8.4 (SD 7.5), and the median HADS-A score was 2.0 (interquartile range (IQR) 0.3–5.0).

### 3.2. Prevalence of Anxiety Disorders

Of 65 patients, 13 (20%) had one or more diagnoses of an anxiety disorder, and 10 (15%) had a diagnosis of a current depressive episode identified by the reference standard MINI interview. Of the patients with an anxiety disorder, two (3%) were diagnosed with a panic disorder, two (3%) with social phobia, and two (3%) with PTSD. In 10 patients (15%), a diagnosis of specific phobia was found, but only one of these patients had an encounter with the object or situation of their phobia in the past two weeks and was therefore included in the analysis.

### 3.3. Interrater Reliability and Performance

Of 10 random MINI interviews performed both by the medical resident and the psychiatrist, three cases were discussed due to a discrepancy in the diagnosis. The consensus was reached after discussion in two cases. In the third case, a depressive episode was diagnosed by both the medical resident and the psychiatrist, but no consensus on the timing of the episode was reached because different information was given by the participant in the interviews. Inter-rater reliability was found to be Kappa 0.82 (*p* < 0.001).

Confirmatory factor analysis showed a CFI of 0.581 and RMSEA of 0.131 for the BAI, and a CFI of 0.938 and RMSAE of 0.107 for the HADS-A, indicating that both the BAI and HADS-A are not unidimensional. Cronbach’s alpha was 0.86 for the BAI and 0.82 for the HADS-A.

### 3.4. Diagnostic Accuracy of the BAI and HADS-A

Cross-tabulation of the BAI and HADS-A by the MINI is presented in Table 2. The ROC curve for the BAI showed good diagnostic accuracy with an AUC of 0.95 (95% confidence interval (CI) 0.89; 1.00) (Supplementary Figure S1). The optimal cut-off value was ≥13 with a sensitivity of 100% and a specificity of 85% (Table 3). Due to nine false-positive cases, the PPV was 0.36. These nine cases scored high on somatic symptoms of anxiety measured by the BAI such as difficulty breathing, unsteadiness, wobbliness of legs, sweating, and dizziness. With no false-negative cases, the NPV was 1.00. The LR+ was 6.7 and the LR- was 0.

The ROC curve for the HADS-A also showed good diagnostic accuracy, with an AUC of 0.95 (95%CI 0.85; 1.00) (Supplementary Figure S2). The optimal cut-off value was ≥10, with a sensitivity of 80% and a specificity of 100%. There were no false-positive cases, making the PPV 1.00, and with one false negative case, the NPV was 0.98. The LR+ was undefined due to division by zero, and the LR- was 0.2 (Table 3).

## 4. Discussion

The aim of our study was to validate the diagnostic accuracy of two widely used screening tools for anxiety, the BAI and HADS-A, in detecting clinically relevant symptoms of anxiety in hemodialysis patients. To the best of our knowledge, there are only a few studies that have validated the HADS-A and no studies that have validated the BAI in this population. Our results showed that the BAI and HADS-A had a similar discriminative power to detect clinically relevant anxiety in hemodialysis patients, with an optimal cut-off value for the BAI of ≥13 and for the HADS-A of ≥10.

We found a prevalence of anxiety disorders with the MINI of 20%, including all specific phobias. This was comparable to a recent systematic review and meta-analysis that found a prevalence of 19% of anxiety disorders among chronic kidney disease patients, but less than a comparable validation study in hemodialysis patients that found a prevalence of 46% [5,23]. In a validation study by Cukor and colleagues, a poor predictive power of the HADS-A was found, in contrast to our findings [23]. Similar to our results, specific phobias were the most common diagnosis in this study, with a prevalence of 27%. It is possible that we found a better performance of the HADS-A in our study because we excluded patients with specific phobias who did not have an encounter with the topic of their phobia in the past two weeks. Validation of screening tools for specific phobias is complicated, as these patients may not experience anxiety related to their phobia in the same timeframe in which the screening tool was administered.

Optimal cut-off values for the BAI vary in the literature, and range from ≥10 in the general population and ≥12 to ≥16 in other chronically ill patient populations or older adults [16,17,33]. This variety in cut-off values for the BAI could be attributable to differences in patient characteristics, but could also be due to an overlap between anxiety symptoms and the symptoms of chronic disease and depression [6,15,19]. This overlap with the symptoms of other conditions could be a reason for our finding of the relatively poor PPV (0.36) of the BAI in our cohort. On the other hand, the NPV of 1.00 of the BAI using a cut-off value of ≥13 in our cohort suggested that it might be a good instrument to rule out anxiety disorders in hemodialysis patients.

Suggested cut-off scores for the HADS-A varied from ≥6 in a dialysis cohort, ≥7 in Parkinson’s disease, and ≥8 in a review of patients from both the general population as well as in medical settings [11,17,22]. We found a higher cut-off score of ≥10 with a high positive predictive value of 1.00 and a high negative predictive value of 0.98. This suggested that the HADS-A was good at both detecting anxiety disorders and also at ruling them out. The relatively high cut-off score we found might have been due to the presence of symptoms related to general distress common in chronically ill patients, instead of symptoms related to an actual anxiety disorder [34].

As the main goal of our study was to validate instruments in order to screen for clinically relevant anxiety in dialysis patients, it was more important to choose cut-off scores based on their ability to capture all the respondents with anxiety disorders (high sensitivity and high NPV) in exchange for an increased chance of getting a false-positive score (lower PPV). The burden of a dialysis patient undergoing one psychiatric consultation in which no anxiety disorders are identified is likely to be less harmful than missing a patient who is in actual need of psychiatric treatment and who is at risk of poorer health outcomes associated with the presence of anxiety disorders [3,4,6]. Where PPV and NPV depend on the prevalence of a disease in a certain population, likelihood ratios do not. The likelihood ratio is a powerful measure of the diagnostic accuracy of a test, and indicates how much that result will raise or lower the probability of disease [35]. For the BAI, we found an LR+ of 6.7, which corresponded to a moderate increase in the likelihood of having a disease after scoring ≥ 13, and an LR- of zero, which corresponded to a large decrease in the likelihood of disease after scoring < 13. The LR+ of the HADS-A could not be calculated due to division by zero, which corresponded to a large increase in the likelihood of disease after scoring ≥ 10, and the LR- of 0.2 corresponded to a small to moderate decrease after scoring < 10. Therefore, screening with the BAI or the HADS-A is useful to detect hemodialysis patients in need of further psychiatric assessment and possible treatment of their anxiety symptoms, although the HADS-A would be the preferred screening tool over the BAI due to a high NPV and LR+ without compromising on the PPV.

Multiple strengths of this study can be identified. This was the first study to validate two widely used screening tools for anxiety disorders in hemodialysis patients. We included patients from eight urban dialysis centers with a multiethnic population, which increased the generalizability of the results. In addition, the exclusion of patients with specific phobias who had no exposure to the specific situation or object related to the phobia in the past two weeks in the analysis was a strength, as these patients were likely to not have experienced symptoms of anxiety related to their diagnosis that could be measured by the screening tool. Furthermore, there was inter-rater reliability of almost perfect agreement.

Limitations of this study included the limited sample size and relatively low number of diagnoses of anxiety disorders that were included in the analysis, which could have decreased the generalizability. Cukor and colleagues found that 46% of patients met the criteria of a DSM-IV diagnosis of anxiety disorder in a single urban hemodialysis center, compared to 20% in our study [23]. It is possible that anxiety disorders were not that prevalent in the study population of DIVERS-II, or that there was a selection bias in patients who were willing to participate in the DIVERS-II study or in a diagnostic interview on anxiety due to avoidant coping style, which might have reduced the generalizability of the results. Second, as we did not have a diagnosis of GAD with the MINI in our study population, we cannot draw conclusions about diagnosing GAD with the BAI or HADS-A in hemodialysis patients. Third, three of the MINI interviews were conducted by telephone instead of face-to-face due to COVID-19 measures. Although this might have affected the accuracy of diagnosis, we did not expect a large impact on the results due to the structured nature of the MINI. Fourth, there was no Dutch translation of the MINI available that was compatible with the DSM-5 at the time of data acquisition. The differences between the DSM-IV and DSM-5 relevant to this paper are that PTSD is excluded from anxiety disorders and agoraphobia is separated from panic disorder in the DSM-5 in comparison to the DSM-IV [36]. The use of the DSM-IV could limit the clinical utility of our results and comparability with future validation studies. Fifth, the CFA showed that the BAI and the HADS are not unidimensional. An extensive exploration of the dimensions of both the HADS and BAI are discussed elsewhere, in which both the possible symptom dimensions of anxiety are discussed, as well as the unidimensional performance of different self-reported questionnaires in the dialysis population [37,38].

## 5. Conclusions

In conclusion, given the mentioned limitations, both the BAI and the HADS-A seemed to be valid and quick screening instruments for detecting clinically relevant anxiety in hemodialysis patients that can be easily administered in routine dialysis care. The suggested cut-off value for the BAI was ≥13 and for the HADS-A was ≥10 in this population, based on our limited data. The exclusion of somatic symptoms of anxiety in the HADS-A, the lower number of items, and the high predictive value might make it more useful in clinical practice than the BAI. As diagnostic accuracy of screening tools varies between settings and patient groups, further validation of anxiety screening tools in larger cohorts of hemodialysis populations from different health systems is needed to investigate the robustness of our findings and to strengthen the current evidence on this topic. Although our study had several limitations, our study added to the current literature, as this was one of the first studies to validate screenings tools for anxiety in the dialysis population, which can be useful to timely recognize and treat anxiety in dialysis patients and improve their mental health and quality of life.

## Figures and Tables

**Table 1:** Baseline characteristics of 65 hemodialysis patients.

Characteristic	All Patients (*n* = 65)
**Demographic**	
Age (years)	66 ± 13
Male sex (*n* (%))	45 (69%)
Immigrant *	18 (28%)
Country of birth	
The Netherlands	51 (79%)
**Social**	
Married/in a relationship	26 (40%)
Has children	44 (68%)
Education **	
Low	16 (25%)
Middle	31 (48%)
High	17 (27%)
Employed	8 (12%)
**Renal and dialysis**	
Dialysis vintage (months)	23 [8–39]
Primary kidney disease	
Renal vascular disease	14 (22%)
Diabetic nephropathy	16 (25%)
Glomerulonephritis	11 (17%)
Other	21 (32%)
Kt/V_urea_ at baseline	3.7 ± 1.1
On the waiting list for kidney transplant	17 (36%)
Residual diuresis of ≥100 mL/24 h	47 (72%)
**Clinical**	
Davies comorbidity score	
Low comorbidity	15 (23%)
Moderate comorbidity	40 (62%)
High comorbidity	10 (15%)
Comorbid conditions	
Diabetes mellitus	29 (45%)
Cardiovascular disease ***	50 (77%)
Laboratory	
Hemoglobin (g/dL)	10.8 ± 1.3
Phosphate (mg/dL)	5.0 ± 1.6
Albumin (g/L)	3.8 ± 0.5
Parathyroid hormone (pg/mL)	48 ± 37
**Psychiatric**	
Psychiatric diagnosis in medical history None Major depressive disorder Anxiety disorder Other	55 (85%) 4 (6%) 0 (0%) 7 (11%)
Anxiety and depressive symptoms	
HADS total score	8.7 ± 6.0
Anxiety symptoms	
HADS-A score	2.0 [0.3–5.0]
BAI score	8.4 ± 7.5
Depressive symptoms	
HADS-D score	5.4 ± 3.2
BDI-II score	13.2 ± 7.7
Current psychotherapy	1 (2%)
Antidepressant use SSRI SNRI Tricyclic	10 (16%) 3 (5%) 0 (0%) 7 (11%)

Note: Values are presented as mean ± standard deviation, median [interquartile range], or frequency (percentage). Abbreviations: BAI, Beck Anxiety Inventory; BDI-II, Beck Depression Inventory; HADS, Hospital Anxiety and Depression Scale; HADS-A, Hospital Anxiety and Depression Scale—Anxiety subscale; HADS-D, Hospital Anxiety and Depression Scale—Depression subscale; SNRI, serotonin and norepinephrine reuptake inhibitors; SSRI, selective serotonin reuptake inhibitor. * Immigrant status was based on the country of birth of the patient or on the country of birth of one or both biological parents. ** Education: low = primary education, middle = secondary education, high = higher professional education and university. *** Cardiovascular disease = acute coronary syndrome, angina pectoris, percutaneous coronary angioplasty, coronary artery bypass surgery, heart failure, peripheral arterial vascular disease, stroke, hypertension.

**Table 2:** (a) Cross-tabulation of the BAI according to the results of the MINI. (b) Cross-tabulation of the HADS-A according to the results of the MINI.

	No Anxiety Diagnosis (MINI)	Anxiety Diagnosis (MINI)	Total
(a)			
AI < 13	51 (78%)	0 (0%)	51 (78%)
BAI ≥ 13	9 (14%)	5 (8%)	14 (22%)
Total	60 (92%)	5 (8%)	65 (100%)
(b)			
HADS-A < 10	60 (92%)	1 (2%)	61 (94%)
HADS-A ≥ 10	0 (0%)	4 (6%)	4 (6%)
Total	60 (92%)	5 (8%)	65 (100%)

Abbreviations: BAI, Beck Anxiety Inventory; HADS-A, Hospital Anxiety and Depression Scale—Anxiety subscale; MINI, Mini International Neuropsychiatric Inventory.

**Table 3:** Characteristics of the BAI and HADS-A.

Screening Tool	AUC	Optimal Cut-Off	Sensitivity	Specificity	PPV	NPV	LR+	LR-
BAI	95%	13	1.00	0.85	0.36	1.00	6.7	0
HADS-A	95%	10	0.80	1.00	1.00	0.98	- *	0.2

Abbreviations: AUC, area under the curve; BAI, Beck Anxiety Inventory; HADS-A, Hospital Anxiety and Depression Scale—Anxiety subscale; LR+, positive likelihood ratio; LR-, negative likelihood ratio; NPV, negative predictive value; PPV, positive predictive value. * Divided by zero.

## Data Availability

The data presented in this study are available upon reasonable request from the corresponding author. The data are not publicly available due to privacy reasons.

## Supplementary Materials

The following are available online at https://www.mdpi.com/article/10.3390/jpm12071077/s1, Table S1: Table of participating dialysis centes and corresponding cities, Figure S1: ROC curve of the Beck Anxiety Inventory in 65 hemodialysis patients, Figure S2: ROC curve of the Hospital Anxiety and Depression Scale—Anxiety subscale in 65 hemodialysis patients.

## References

[1] (1994). Diagnostic and Statistical Manual of Mental Disorders: DSM-IV.

[2] Cohen S.D., Cukor D., Kimmel P.L. (2016). Anxiety in Patients Treated with Hemodialysis. Clin. J. Am. Soc. Nephrol..

[3] Preljevic V.T., Osthus T.B., Os I., Sandvik L., Opjordsmoen S., Nordhus I.H., Dammen T. (2013). Anxiety and depressive disorders in dialysis patients: Association to health-related quality of life and mortality. Gen. Hosp. Psychiatry.

[4] Schouten R.W., Haverkamp G.L., Loosman W.L., Chandie Shaw P.K., van Ittersum F.J., Smets Y.F.C., Vleming L.J., Dekker F.W., Honig A., Siegert C.E.H. (2019). Anxiety Symptoms, Mortality, and Hospitalization in Patients Receiving Maintenance Dialysis: A Cohort Study. Am. J. Kidney Dis..

[5] Huang C.W., Wee P.H., Low L.L., Koong Y.L.A., Htay H., Fan Q., Foo W.Y.M., Seng J.J.B. (2021). Prevalence and risk factors for elevated anxiety symptoms and anxiety disorders in chronic kidney disease: A systematic review and meta-analysis. Gen. Hosp. Psychiatry.

[6] Kimmel P.L., Cukor D. (2019). Anxiety Symptoms in Patients Treated with Hemodialysis: Measurement and Meaning. Am. J. Kidney Dis..

[7] Inker L.A., Astor B.C., Fox C.H., Isakova T., Lash J.P., Peralta C.A., Kurella Tamura M., Feldman H.I. (2014). KDOQI US commentary on the 2012 KDIGO clinical practice guideline for the evaluation and management of CKD. Am. J. Kidney Dis..

[8] Pascoe M.C., Thompson D.R., Castle D.J., McEvedy S.M., Ski C.F. (2017). Psychosocial Interventions for Depressive and Anxiety Symptoms in Individuals with Chronic Kidney Disease: Systematic Review and Meta-Analysis. Front. Psychol..

[9] Reavell J., Hopkinson M., Clarkesmith D., Lane D.A. (2018). Effectiveness of Cognitive Behavioral Therapy for Depression and Anxiety in Patients With Cardiovascular Disease: A Systematic Review and Meta-Analysis. Psychosom. Med..

[10] Richards S.H., Anderson L., Jenkinson C.E., Whalley B., Rees K., Davies P., Bennett P., Liu Z., West R., Thompson D.R. (2017). Psychological interventions for coronary heart disease. Cochrane Database Syst. Rev..

[11] Preljevic V.T., Osthus T.B., Sandvik L., Opjordsmoen S., Nordhus I.H., Os I., Dammen T. (2012). Screening for anxiety and depression in dialysis patients: Comparison of the Hospital Anxiety and Depression Scale and the Beck Depression Inventory. J. Psychosom. Res..

[12] Martin C.R., Thompson D.R. (2002). The hospital anxiety and depression scale in patients undergoing peritoneal dialysis: Internal and test–retest reliability. Clin. Eff. Nurs..

[13] Beck A.T., Epstein N., Brown G., Steer R.A. (1988). An inventory for measuring clinical anxiety: Psychometric properties. J. Consult. Clin. Psychol..

[14] Beck A.T., Epstein N., Brown G., Steer R. (1993). Beck Anxiety Inventory Manual.

[15] Balsamo M., Cataldi F., Carlucci L., Fairfield B. (2018). Assessment of anxiety in older adults: A review of self-report measures. Clin. Interv. Aging.

[16] Dennis R.E., Boddington S.J., Funnell N.J. (2007). Self-report measures of anxiety: Are they suitable for older adults?. Aging Ment. Health.

[17] Leentjens A.F., Dujardin K., Marsh L., Richard I.H., Starkstein S.E., Martinez-Martin P. (2011). Anxiety rating scales in Parkinson’s disease: A validation study of the Hamilton anxiety rating scale, the Beck anxiety inventory, and the hospital anxiety and depression scale. Mov. Disord..

[18] Leyfer O.T., Ruberg J.L., Woodruff-Borden J. (2006). Examination of the utility of the Beck Anxiety Inventory and its factors as a screener for anxiety disorders. J. Anxiety Disord..

[19] Muntingh A.D., van der Feltz-Cornelis C.M., van Marwijk H.W., Spinhoven P., Penninx B.W., van Balkom A.J. (2011). Is the Beck Anxiety Inventory a good tool to assess the severity of anxiety? A primary care study in the Netherlands Study of Depression and Anxiety (NESDA). BMC Fam. Pract..

[20] Kabacoff R.I., Segal D.L., Hersen M., Van Hasselt V.B. (1997). Psychometric properties and diagnostic utility of the Beck Anxiety Inventory and the State-Trait Anxiety Inventory with older adult psychiatric outpatients. J. Anxiety Disord..

[21] Zigmond A.S., Snaith R.P. (1983). The hospital anxiety and depression scale. Acta Psychiatr. Scand..

[22] Bjelland I., Dahl A.A., Haug T.T., Neckelmann D. (2002). The validity of the Hospital Anxiety and Depression Scale. An updated literature review. J. Psychosom. Res..

[23] Cukor D., Coplan J., Brown C., Friedman S., Newville H., Safier M., Spielman L.A., Peterson R.A., Kimmel P.L. (2008). Anxiety disorders in adults treated by hemodialysis: A single-center study. Am. J. Kidney Dis..

[24] Nadort E., Schouten R.W., Dekker F.W., Honig A., van Oppen P., Siegert C.E.H. (2019). The (cost) effectiveness of guided internet-based self-help CBT for dialysis patients with symptoms of depression: Study protocol of a randomised controlled trial. BMC Psychiatry.

[25] Cohen J.F., Korevaar D.A., Altman D.G., Bruns D.E., Gatsonis C.A., Hooft L., Irwig L., Levine D., Reitsma J.B., de Vet H.C.W. (2016). STARD 2015 guidelines for reporting diagnostic accuracy studies: Explanation and elaboration. BMJ Open.

[26] Sheehan D.V., Lecrubier Y., Sheehan K.H., Amorim P., Janavs J., Weiller E., Hergueta T., Baker R., Dunbar G.C. (1998). The Mini-International Neuropsychiatric Interview (M.I.N.I.): The development and validation of a structured diagnostic psychiatric interview for DSM-IV and ICD-10. J. Clin. Psychiatry.

[27] Van Dijk P.C., Jager K.J., de Charro F., Collart F., Cornet R., Dekker F.W., Gronhagen-Riska C., Kramar R., Leivestad T., Simpson K. (2001). Renal replacement therapy in Europe: The results of a collaborative effort by the ERA-EDTA registry and six national or regional registries. Nephrol. Dial. Transplant..

[28] Davies S.J., Phillips L., Naish P.F., Russell G.I. (2002). Quantifying comorbidity in peritoneal dialysis patients and its relationship to other predictors of survival. Nephrol. Dial. Transplant..

[29] Arifin W.N. (2022). Sample Size Calculator. http://wnarifin.github.io.

[30] Landis J.R., Koch G.G. (1977). An application of hierarchical kappa-type statistics in the assessment of majority agreement among multiple observers. Biometrics.

[31] Rosseel Y. (2012). Lavaan: An R Package for Structural Equation Modeling. J. Stat. Softw..

[32] Krzanowski W.J., Hand D.J. (2009). ROC Curves for Continuous Data.

[33] De Lima Osório F., Crippa J.A.S., Loureiro S.R. (2011). Further psychometric study of the Beck Anxiety Inventory including factorial analysis and social anxiety disorder screening. Int. J. Psychiatry Clin. Pract..

[34] Hudson J., Chilcot J., Goldsmith D., Covic A., Spaak J. (2015). Psychological Distress in Physical Long-Term Conditions. Cardio-Renal Clinical Challenges.

[35] Hayden S.R., Brown M.D. (1999). Likelihood ratio: A powerful tool for incorporating the results of a diagnostic test into clinical decisionmaking. Ann. Emerg. Med..

[36] Kupfer D.J. (2015). Anxiety and DSM-5. Dialogues Clin. Neurosci..

[37] Schouten R.W., Harmse V.J., Dekker F.W., van Ballegooijen W., Siegert C.E.H., Honig A. (2019). Dimensions of Depressive Symptoms and Their Association with Mortality, Hospitalization, and Quality of Life in Dialysis Patients: A Cohort Study. Psychosom. Med..

[38] Schouten R.W., Nadort E., Harmse V., Honig A., van Ballegooijen W., Broekman B.F.P., Siegert C.E.H. (2020). Symptom dimensions of anxiety and their association with mortality, hospitalization and quality of life in dialysis patients. J. Psychosom. Res..

