# Supplementary material for: Validation of Two Screening Tools for Anxiety in Hemodialysis Patients
Source: J Pers Med. 2022 Jun 30;12(7):1077. doi: 10.3390/jpm12071077 (PMC9318791; doi:10.3390/jpm12071077)

Supplementary Material

Table S1: Table of participating dialysis centers and corresponding cities.

**Dialysis centers**	**City**
OLVG West	Amsterdam
Haaglanden Medisch Centrum, Westeinde	The Hague
HagaZiekenhuis Dialyse Centrum Zoetermeer	Zoetermeer
Spaarne Gasthuis Haarlem Zuid<br>Spaarne Gasthuis Hoofddorp<br>Spaarne Gasthuis Velsen Noord	Haarlem<br>Hoofddorp<br>Velsen
Tergooi Ziekenhuis<br>Dialysecentrum ‘t Gooi	Hilversum<br>Hilversum

Figure S1: ROC curve of the Beck Anxiety Inventory in 65 hemodialysis patients.

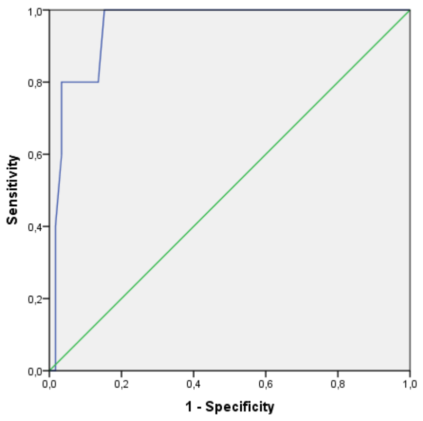

Figure S2: ROC curve of the Hospital Anxiety and Depression Scale – Anxiety subscale in 65 hemodialysis patients.

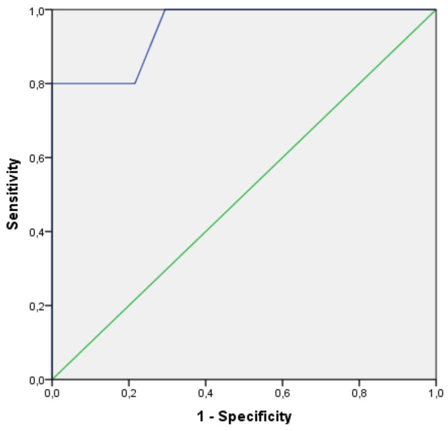

Supplement: Supplementary file 1 [file jpm-12-01077-s001.zip › jpm-1660101-supplementary.pdf]
